# Host-Pathogen O-Methyltransferase Similarity and Its Specific Presence in Highly Virulent Strains of *Francisella tularensis *Suggests Molecular Mimicry

**DOI:** 10.1371/journal.pone.0020295

**Published:** 2011-05-26

**Authors:** Mia D. Champion

**Affiliations:** Division of Pathogen Genomics, Translational Genomics Research Institute, Arizona, United States of America; Université Paris-Sud, France

## Abstract

Whole genome comparative studies of many bacterial pathogens have shown an overall high similarity of gene content (>95%) between phylogenetically distinct subspecies. In highly clonal species that share the bulk of their genomes subtle changes in gene content and small-scale polymorphisms, especially those that may alter gene expression and protein-protein interactions, are more likely to have a significant effect on the pathogen's biology. In order to better understand molecular attributes that may mediate the adaptation of virulence in infectious bacteria, a comparative study was done to further analyze the evolution of a gene encoding an o-methyltransferase that was previously identified as a candidate virulence factor due to its conservation specifically in highly pathogenic *Francisella tularensis* subsp. *tularensis* strains. The o-methyltransferase gene is located in the genomic neighborhood of a known pathogenicity island and predicted site of rearrangement. Distinct o-methyltransferase subtypes are present in different *Francisella tularensis* subspecies. Related protein families were identified in several host species as well as species of pathogenic bacteria that are otherwise very distant phylogenetically from Francisella, including species of Mycobacterium. A conserved sequence motif profile is present in the mammalian host and pathogen protein sequences, and sites of non-synonymous variation conserved in Francisella subspecies specific o-methyltransferases map proximally to the predicted active site of the orthologous human protein structure. Altogether, evidence suggests a role of the *F. t.* subsp. *tularensis* protein in a mechanism of molecular mimicry, similar perhaps to Legionella and Coxiella. These findings therefore provide insights into the evolution of niche-restriction and virulence in Francisella, and have broader implications regarding the molecular mechanisms that mediate host-pathogen relationships.

## Introduction

Bacterial human pathogens, many of which were at one time easily treatable with antibiotics, have re-emerged within the last couple of decades as highly infectious public health threats, and in some cases, are also potential agents for use as biological weapons. This trend in large part is due to the expanding antibiotic resistance profile of these pathogens, making infections increasingly harder to treat with multiple classes of drugs. In addition to the growing threat of pan-resistance, most bacterial pathogens are highly infectious and many are transmissible when aerosolized and inhaled by mammalian hosts. These include bacterial pathogens such as *Coxiella burnetii*, *Legionella pneumophila*, *Mycobacterium tuberculosis*, and *Francisella tularensis* ; all of which cause severely debilitating diseases that can lead to fatality, especially in immunocompromised humans. Although *Francisella tularensis* is likely better known for its classification as a potential bacterial biological weapon, a recent report of erythromycin resistance in *Francisella tularensis* also emphasizes the relevance of this organism as a model for studies of adaptive biological processes that enable otherwise mildly infectious environmental bacteria to adapt to specific hosts, and become highly virulent pathogens [1], [2].

Although many human bacterial pathogens may share similar lifestyles and carry genomes that are roughly the same size, they are not close phylogenetic relatives. In the case of *Francisella tularensis*, previous studies of 16S rRNA phylogenies suggests only distant relations to other human pathogens, such as *Coxiella burnetii* and *Legionella pneumophila* [3]. These studies also suggest that *F. tularensis* is a sister clade with arthropod endosymbionts like *Wolbachia persica* [3]. Additional sequence similarity analysis, including consensus sequence signatures, identified Francisella as a member of the γ subclass of proteobacteria [3]. *Francisella tularensis* is a gram-negative, facultative, intracellular bacterium and virulent isolates are the etiological cause of tularemia, a severely debilitating and occasionally fatal disease in humans. Tularemia occurs almost exclusively in the Northern Hemisphere; America, Japan, Switzerland, Sweden, Russia and the Mediterranean parts of the world. The severity of the disease depends both on the route of infection and the potency of the Francisella subspecies [4]. *F.t.* subsp *tularensis* is the most pathogenic subspecies of Francisella and transmission to humans typically occurs via inhalation or ingestion of contaminated foods or water. Aerosolization of the bacteria can occur by disruption of small animal carcasses and, in the case of the *tularensis* subspecies, as few as 1–10 cells can be lethal [5], [6]. There are numerous host species that are susceptible to Francisella, however, common secondary hosts or reservoirs are terrestrial and aquatic mammals such as: ground squirrels, rabbits, voles, muskrats, and beavers. Transmission also typically occurs by entry of the bacteria through skin abrasions or sites of bites from an arthropod vector, such as: ticks, *Dermacentor reticulates*, *Ixodes ricinus*, biting flies, and *Aedes spp*, *Culex spp* (mosquitos) [6].

Presently, there are four accepted subspecies of *Francisella tularensis*: *Francisella tularensis* subsp. *tularensis*, *Francisella tularensis* subsp. *holarctica*, *Francisella tularensis* subsp. *novicida* and *Francisella tularensis* subsp. *mediasiatica*. Previous phylogenetic studies have examined the relationships between the different subspecies of Francisella and have provided evidence supporting the more recent divergence of the *F. tularensis* subsp. *holarctica* (Biovar Type B) lineage in comparison to the *F. tularensis* subsp. *tularensis* (Biovar Type A) lineage [4], [7]. The more basal phylogenetic positioning of the *holarctica* FSC022 japonica strain in relation to other Type B strains suggests that the *holarctica* lineage originated in Asia, and is distinct from the radiation lineage found throughout the Northern Hemisphere. There are two known distinct subtypes of the *F. tularensis* subsp. *tularensis* (Type A) lineage, Types A.I and A.II [8], [9], [10] and emerging evidence that subclades of these two subtypes may exist. The highly virulent Type A clade is found in North America, whereas the Type B lineage is the primary cause of Tularemia in Europe, and is associated with more mild clinical symptoms [7], [11]. The *F. tularensis* subsp. *mediasiatica* isolate is geographically restricted to the central Asia region and presents clinically very similarly to the *holarctica* subspecies [2]. However, whole-genome phylogenetic studies have shown that the *mediasiatica* lineage clusters more closely to the *tularensis* subspecies [2], [12].

Similar to many other bacterial pathogens (e.g., MRSA, *Mycobacterium tuberculosis*, *Bacillus anthracis*, *Yersinia pestis*), sequence analysis of *F. tularensis* genomes provides evidence of the overall clonal nature of these strains. Whole-genome comparative studies of many gram-negative bacterial pathogens, including *F. tularensis* have reported greater than 95% overall sequence similarity between representatives of distinct phylogenetic lineages. The evolution of pathogenic bacterial species from nonpathogenic ancestors is therefore marked by relatively small changes in the overall gene content. Pathoadaptive mutations include acquisition of genes mediating virulence, typically by horizontal gene transfer via plasmids or pathogenicity islands, modifications of gene expression, or gene loss events that enable the bacteria to better adapt to the host-niche or become a more potent pathogen [13].

Recent comparative studies using whole-genome sequences have provided support for a proposed evolutionary process of Francisella pathogenicity [2], [12]. Ancestral Francisella populations are geographically disperse and environmentally free-living strains, that undergo recombination. The differentiation of the *F. tularensis* lineage from these ancestral populations enabled invasion of a novel and more restricted host-niche: The human. Previous studies have shown an increased presence of specific families of transposable elements in the more pathogenic species of Francisella, thus resulting in higher frequencies of genome rearrangement events, resulting in both gene loss and duplication events. Rearrangement events enabled loss of any genes whose function was no longer necessary in a nutritionally rich host-niche [2], [12]. Interestingly, the less virulent and more recently emerged *holarctica* subspecies is presently experiencing the highest level of genome decay, which may signify an increasing adaptation and dependence on a restricted mammalian host niche [12]. *F. tularensis* subsps. *tularensis* , *holarctica*, and *mediasiatica* are not metabolically competent and most, if not all, pathways for amino acid synthesis are inactivated in these subspecies [12], [14], [15]. Although the *F.t. novicida* lineage is considered a F.*tularensis* subspecies, there is debate regarding whether it should be classified as a separate species of Francisella. *F.t. novicida* isolates and isolates of *F. philomiragia*, the only accepted species in addition to *F. tularensis* that belongs to the genus Francisella, are from environmental samples and are metabolically competent. Unlike the highly virulent *tularensis* subspecies, *F.t. novicida* and *F. philomiragia* are not potent human pathogens and have only been known to cause disease primarily in immuno-compromised individuals or victims of near drowning [11]. Overall, the more pathogenic *tularensis* and *holarctica* subspecies of Francisella have undergone genome erosion processes associated with host-restriction [2], [12].

Gain of gene function promoting an aggressive pathogenic life-style has also played a role in the pathoadaptation of *F. tularensis*. The acquisition and duplication of a pathogenicity island in the more pathogenic strains is evident from comparisons of different subspecies of Francisella [16]. Genomes from certain isolates belonging to the more pathogenic *tularensis* and *holarctica* lineages share conserved ORFs that are differentially disrupted in other subspecies or subclades, and include several genes predicted to encode membrane proteins and proteins of unknown function, as well as an adenosine deaminase [12], [15]. Of particular interest was the finding that all strains of the highly virulent *tularensis* subspecies carry the same allele of a gene predicted to encode a specialized o-methyltransferase, that is differentially disrupted or absent in all other Francisella subspecies [12], [15]. This observation led to the hypothesis that conservation of the intact o-methyltransferase gene specifically in the *F.t.* subsp. *tularensis* lineage 1) defines pathogen fitness, or potency, and is maintained and propagated in the population 2) is conserved with other highly virulent, and phylogenetically distant pathogens with similar lifestyles 3) was independently acquired by more than one lineage, either by functional convergence favoring select sequence state changes or by mechanisms of gene-transfer. The former mechanism of convergent evolution between non-clonal, or rather distantly related bacteria is expected to lead to highly similar traits whereas the latter mechanism of signature transfer should be evident by the presence of nearly identical sequence signature attributes.

It is reported here that the *F.t.* subsp. *tularensis* o-methyltransferase sequence is conserved in other bacteria including known human pathogens. Orthologs of the o-methyltransferase sequences were also identified in known host organisms, including human. Analysis of predicted secondary structures of the subspecies specific o-methyltransferase alleles and comparison to the orthologous human protein structure suggests a functional role for the highly conserved subtype in virulent strains of Francisella. These findings in light of recent reports describing the role of o-methyltransferase in mechanisms of molecular mimicry in other aerosolized pathogens, such as *Legionella pneumophila* and *Coxiella burnetii*, provides insight into Francisella pathogenicity [17], [18], [19]. And also suggests that perhaps specific human host-pathogen biological pathways are under selective pressure, and even otherwise very distantly related bacteria evolve similar traits that are necessary in order for them to lead a virulent life-style [20].

## Results and Discussion

### Identification of Francisella tularensis subspecies specific o-methyltransferase orthologs in otherwise distant bacteria, including other human pathogens, and host organisms

**Figure 1:**
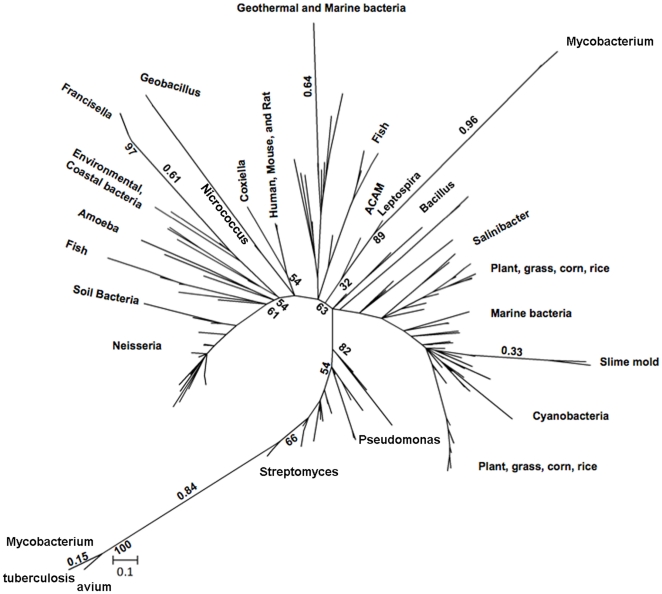
Phylogenetic relationships among o-methyltransferase orthologs. 212 identified orthologs of *Francisella tularensis* subsp. *tularensis* o-methyltransferase. Phylogenetic trees were constructed with protein sequences using the Maximum Likelihood method. Bootstrap values were calculated after 1000 replicates, and the consensus tree is shown. Values of branches clustering select species are shown above the branch and frequencies are proximal to nodes of interest. Francisella o-methyltransferase subtypes phylogenetically cluster with orthologs identified in species of environmental and coastal bacteria (e.g Marnimonas genus). A neighboring cluster shows a tight clustering between the Coxiella and Human, Mouse and Rat o-methyltransferase orthologs.

A protein family analysis pipeline that incorporates algorithms for validation, integration, and presentation of highly conserved protein families at the sequence, structural and functional levels was used to search for orthologs of the highly conserved *F.t.* subsp. *tularensis* (Type A) o-methyltransferase [21]. A composite database of 13,062,032 sequences was queried using the Type A o-methyltransferase translated protein sequence. A Ballast profile was derived from the top 200 protein sequences aligning with the highest blastp alignment similarity and used to refine the gapped blast alignments based on locally conserved segments [22]. DbClustal analysis was done to perform accurate global alignments and generate multiple sequence alignments, further refined by the RASCAL correction algorithm [23], [24]. The Leon algorithm was used to cluster the multiple sequence alignments into distinct protein sequence families based on residue composition and conservation [21]. A second, and less stringent approach was also used to search for protein sequences exhibiting significant similarity to the Type A o-methyltransferase protein sequence (blastp, expectation threshold <1e-11). Interestingly, several sequences belonging to diverse species of Mycobacterium were identified as significantly similar to the highly virulent *F. tularensis* subsp. *tularensis* o-methyltransferase sequence. These 12 Mycobacterium sequences were therefore realigned with the 200 other o-methyltransferase protein sequences. A multiple protein sequence alignment of identified o-methyltransferase orthologs was used to construct a phylogenetic tree using Maximum likelihood Estimation methods (Figure 1).

The *F. tularensis* o-methyltransferase subtypes cluster phylogenetically together with otherwise very distant bacteria. Orthologs were identified in species of the Marnimonas genus, which are gamma-proteobacteria that exist in coastal waters (Figure 1). A neighboring cluster consists of species of distant marine bacteria: Geobacillus and Nicrococcus, together with the more closely related human pathogen Coxiella as well as mammalian o-methyltransferase orthologs. The closest relationship in this specific cluster is between the Coxiella o-methyltransferase orthologs and protein sequences identified from the human, mouse and rat genomes (Figure 1). Also present in the phylogeny are many related o-methyltransferase sequences from other human pathogen species such as Pseudomonas and Streptomyces. SAM dependent methyltransferase orthologs are also present in *Legionella pneumophila*, with the *L. pneumophila Lens* strain ortholog exhibiting the highest sequence similarity to the *Francisella tularensis* subsp. *tularensis* o-methyltransferase (blastp, 6e^−37^). Overall, the Mycobacterium orthologs are more divergent than the rest of the group and are divided into two distinct phylogenetic clusters; one of which is in close relationship with sequences of Streptomyces. Phylogenetic relationships also include many species of fish and marine animals (e.g., salmon, rainbow trout, trichoplorax adherens), as well as plants, grasses, corn and rice (Figure 1). The *F. tularensis* subsp. *tularensis* o-methyltransferase is related to Caffeoyl-CoA o-methyltransferases (EC 2.1.1.104). Generally, Caffeoyl-CoA o-methyltransferases have been best characterized in plant species where they are known bifunctional enzymes that mediate secondary metabolite synthesis [25]. In addition, these enzymes have a known role in lignin biosynthesis, which is an enzyme that plays a pivotal role in cell wall reinforcement during the induced disease resistance response in plants [26], [27].

Underlying the phylogenetic relationship of these related families of o-methyltransferases are several highly conserved sequence blocks and the presence of numerous residue conservation profiles that are suggestive of canonical structural domains. Regions of high protein sequence conservation between the subspecies of Francisella begin with a MAQ consensus sequence, which is also conserved in the plant commensal *Pseudomonas fluorescens* Pf-5 sequence.

### Distinct protein sequence variants are highly conserved in different subspecies of Francisella

**Figure 2:**
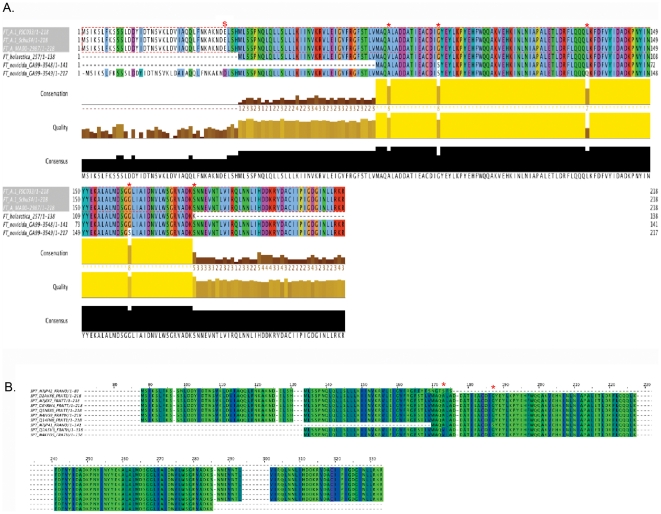
Francisella subspecies specific o-methyltransferase variants. A) Multiple sequence alignment of translated o-methyltransferase sequences exhibit high conservation between representatives of different Francisella subspecies. There is an internal predicted stop codon present in the ORF of the *F.t. novicida* GA99-3549 ortholog (s) and in the ORF of a highly degenerate ortholog present in the *F.t. mediasiatica* isolate (not shown). An orthologous sequence of significant similarity is absent from the *F.philomiragia* species. Additional key amino acid changes discussed throughout the manuscript are labeled (*) B) Comparative gapped alignment is shown with two polymorphisms (*) marked that are predicted to effect the buried index (differential shading) and therefore, the predicted higher level of protein folding.

**Figure 3:**
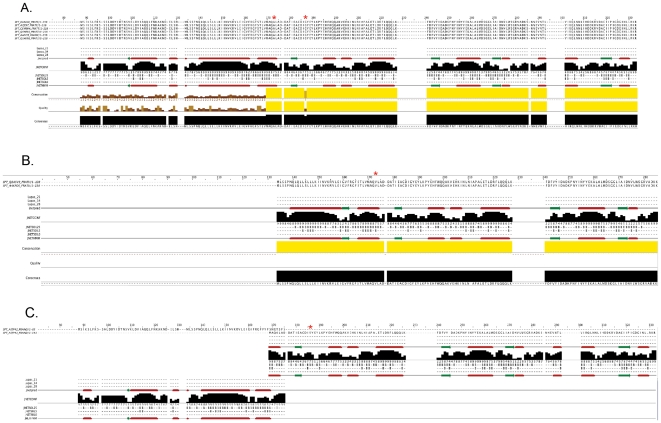
Distinct polymorphisms effect the secondary structure of the predicted protein. A) Multiple o-methyltransferase protein sequences are aligned from the representative *Francisella tularensis* subsp. *tularensis* genomes. The predicted secondary structure is mapped below the multiple alignments and consists of alternating alpha-helices (red tubes) and beta-sheets (green arrows) characteristic of this family of conserved o-methyltransferases. Key amino acids that are nonsynonymous mutations in the other subspecies are indicated with a red asterisk (*). B) Alignment of *Francisella tularensis* subsp. *holarctica* o-methyltransferase orthologs and secondary structure predictions shows that approximately half of the protein structure is missing in comparison to the *Francisella tularensis* subsp. *tularensis* o-methyltransferase. A nonsynonymous amino acid change conserved in the *Francisella tularensis* subsp. *holarctica* subspecies is indicated with a red asterisk (*). C) Alignment of the *Francisella tularensis* subsp. *novicida* GA99-3548 o-methyltransferase sequence encoded by ORFs annotated as two independent loci illustrate the presence of two separate conserved ‘halves’ of the protein disrupted by an internal frameshift mutation. And a nonsynonymous amino acid change conserved in the *Francisella tularensis* subsp. *novicida* subspecies is indicated with a red asterisk (*). The more recent phylogenetic emergence of the *tularensis* lineage in comparison to the *holarctica* and *novicida* lineages suggests that recombination and selection events may have resulted in gain of a more highly conserved o-methyltransferase ORF that promotes virulence in the Type A strains.

Identification of subspecies specific alleles of o-methyltransferase called for further characterization of the different subtypes, and analysis of possible selective pressures that may be driving the evolution of these distinct protein families in specific subspecies of Francisella of varying pathogenicity. The Francisella o-methyltransferase homologs exhibit a high level of protein sequence similarity between representatives of different Francisella subspecies (Figure 2A). An alignment of the translated sequence from representatives of each Francisella subspecies shows a region of high conservation with the exception of two key amino acid sequence mutations (Figure 2A) that are predicted to effect the buried index, and therefore, higher level protein folding (Figure 2B). There is a sequence consensus (MAQ) starting at amino acid M170 that is highly conserved in most subspecies of Francisella (Figure 3). The most significant subspecies specific divergence occurs 5′ of this consensus site.

An alternate start site is predicted for this o-methyltransferase family in *F. tularensis* subsp. *holarctica* strains in comparison to the *novicida* and *tularensis* o-methyltransferase subtypes (Figure 3). Although the highly conserved sequence block 5′ of the MAQ consensus is conserved between *novicida* and *tularensis* strains, there are frameshift mutations present in both strains (GA99-3548 and GA99-3549) of the *F. tularensis* subsp. *novicida* lineage. In *F. tularensis* subsp. *novicida* GA99-3548, there is a frameshift mutation that has resulted in the o-methyltransferase ORF being annotated as two distinct, overlapping loci (FTDG_01299 and FTDG_01300) (Figure 3C). The 5′ in-frame stop codon present in the *F. tularensis* subsp. *novicida* GA99-3549 strain is labeled in Figure 2A and occurs proximal to the predicted start of the *F. tularensis* subsp. *holarctica* o-methyltransferase. Nonsynonymous changes predicted to effect the function of the gene product occur 3′ of the MAQ consensus site. In the *F. tularensis* subsp. *holarctica* subspecies, o-methyltransferase sequences carry a highly conserved amino acid change at V173 (A->V) in comparison to other *tularensis* subspecies sequences (Figure 3B). And *F. tularensis* subsp. *novicida* sequences carry a highly conserved nonsynonomous change at S186 (G->S)(Figure 3C). Three additional *F. tularensis* subsp. *novicida* strains (FTG, U112, and FTE) were also analyzed and found to carry orthologs of low similarity to the Type A o-methyltransferase sequence (Figure S1). The *F. tularensis* subsp. *novicida* FTG strain also carries a second gene copy (FTG_1138) that also encodes a predicted ortholog. Overall, multiple alignments of this sequence with the other Francisella o-methyltransferase proteins shows that much like the *F. tularensis* subsp. *novicida* GA99-3549 strain, this *novicida* FTG variant exhibits an overall high degree of sequence similarity with the *F. tularensis* subsp. *tularensis* o-methyltransferase. However, there are several nonsynonomous amino acid changes throughout the reading frame, including a *novicida* signature S186 (G->S) transition.

A local blast search identified a highly degenerate ortholog present in the *F. tularensis* subsp. *mediasiatica* FSC147 isolate (bp 71560–72125); Exhibiting limited sequence similarity to the homologous region 5′ of the MAQ consensus site in *F. tularensis* subsp. *tularensis*. Sequence divergence in the *F. tularensis* subsp. *mediasiatica* ortholog occurs proximal to the predicted *F. tularensis* subsp. *holarctica* start site, resulting in several downstream in-frame stop codons. Although a *F. philomiragia* predicted o-methyltransferase was identified in blast searches (FTPG_00511), the identified sequence exhibits only ∼25% sequence similarity with the *F. tularensis* subsp. *tularensis* protein and is therefore, not considered to be a true ortholog (data not shown).

**Figure 4:**
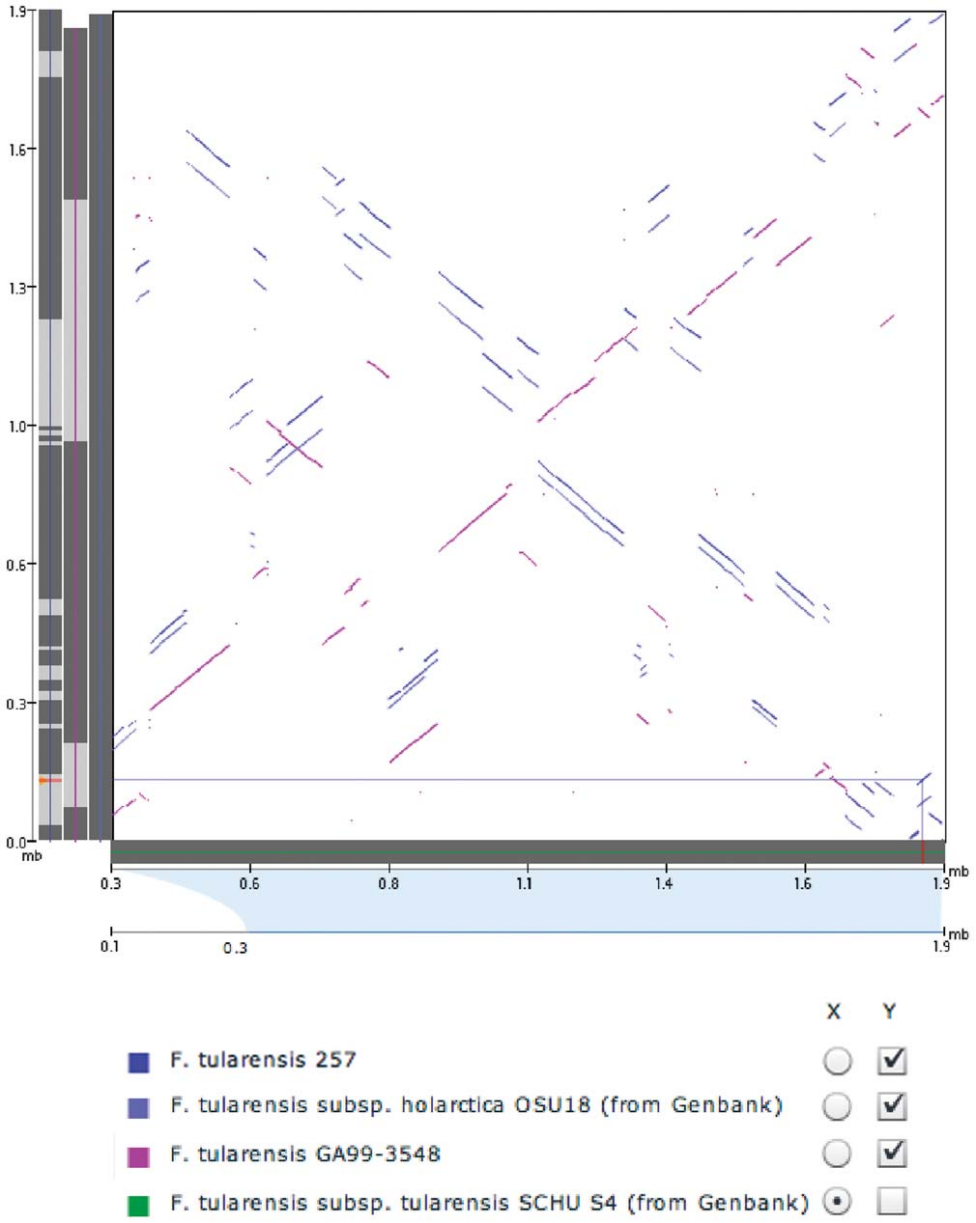
O-methyltransferase orthologs are located proximal to predicted genome rearrangements. A) Dotplot comparison of pairwise alignments between whole-genome sequences from representatives of various Francisella subspecies is shown. The o-methyltransferase ortholog is highlighted on the *Francisella tularensis* subsp. *holarctica* strain 257 in orange (Y-axis) and on the Francisella tularensis subsp tularensis SchuS4 strain (X-axis, reference genome) in red. Blocks of synteny between each of the Francisella genomes compared to the Francisella tularensis subsp tularensis SchuS4 strain are plotted and rearrangements are indicated by breaks in the linearity of the lines and perpendicular orientations.

It seemed relevant to next analyze the genomic context of the gene sequences in order to evaluate how recombination and selection may have influenced the evolution of several distinct o-methyltransferase subtypes that are highly conserved within independent subspecies of Francisella. In *F. tularensis* subsp. *tularensis* , the FTT1766 locus spans a 656 nucleotide region of the genome (bp 1853414–1854070) and is in the genomic neighborhood of one copy of the duplicated pathogenicity islands, comprising a ∼33.9 kb region at around bp 1,800,000 of the genome [14]. Mutations of several genes within the pathogenicity islands have been shown in previous studies to significantly reduce the pathogens ability to survive within amoebae or macrophage hosts. In addition, specific families of Insertion Sequence Elements (ISFtu1 and ISFtu2) are enriched in the more pathogenic species of Francisella and previous studies have provided evidence that IS Element-based genome rearrangement events led to the duplication of the pathogenicity islands in the *F. tularensis* lineage [15]. Genome rearrangements proximal to the o-methyltransferase locus are evident from whole genome alignments and dotplot comparisons between representatives of various Francisella subspecies (Figure 4). The PHI test did not find statistically significant evidence for recombination between gene copies (p = 0.826) [28]. It was thus relevant to assess whether functional differentiation has been mediated by a molecular adaptation process acting on the coding region of the o-methyltransferase locus. Methods which estimate the ratio of the rates of non-synonymous (dN) to synonymous (dS) substitutions can be used to distinguish pseudogene evolution from positive selection; the former is evident by a dN/dS ratio approaching 1 across the length of the gene, rather than several sites within the gene exhibiting a dN/dS ratio >1 [29]. Multiple alignments of the Francisella homologous o-methyltransferase sequences were analyzed for evidence of pseudogene evolution and positive selection [30]. Different models identified several of the same sites, however, the statistical significance was not consistent enough to provide definitive evidence of positive selection.

The proximity of the o-methyltransferase gene to predicted sites of rearrangement that are known to flank a neighboring pathogenicity island supports the hypothesis that recombining ancestral Francisella populations allowed for the differentiation and selection of genomic attributes that likely enabled the invasion of the human host by the *F. tularensis* subsp. *tularensis* lineage. Genes such as this highly conserved o-methyltransferase specific to *F. tularensis* subsp. *tularensis* strains therefore provide key insights into the evolution of pathogenicity and may be functionally important and necessary for virulence. Conservation of distinct o-methyltransferase protein sequence subtypes in different Francisella subspecies suggests differences of functional roles that may influence pathogen potency, fitness or virulence. Characterization of the distinct o-methyltransferase subtypes and their predicted secondary structure seemed necessary in order to better understand differences of probable function, and the relevance of the highly conserved subtype found specifically in the virulent Type A subclade.

### Conservation of the predicted secondary structure of a virulent o-methyltransferase subtype and identification of a motif conservation profile in intracellular pathogens, as well as in host sequences, suggests molecular mimicry

**Figure 5:**
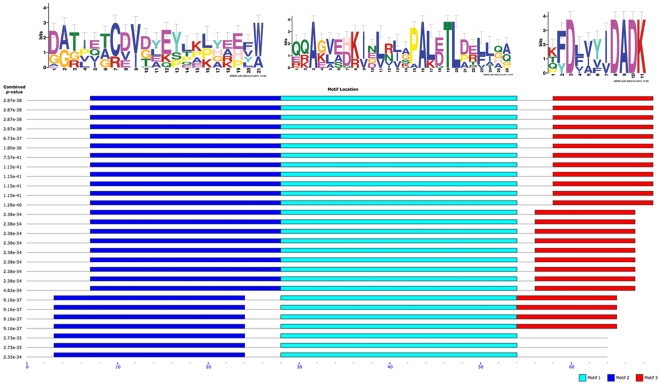
Motif conservation profiles in o-methyltransferase orthologs. Identified MEME motifs with small-sample correction (SSC) are shown above a combined block diagram indicating relative location in each of the aligned sequences. From top to bottom, sequences include Human, Mouse, Rat, Coxiella, Francisella, and Mycobacterium isolates. Combined p-values for the motif profiles are shown next to each sequence.

**Figure 6:**
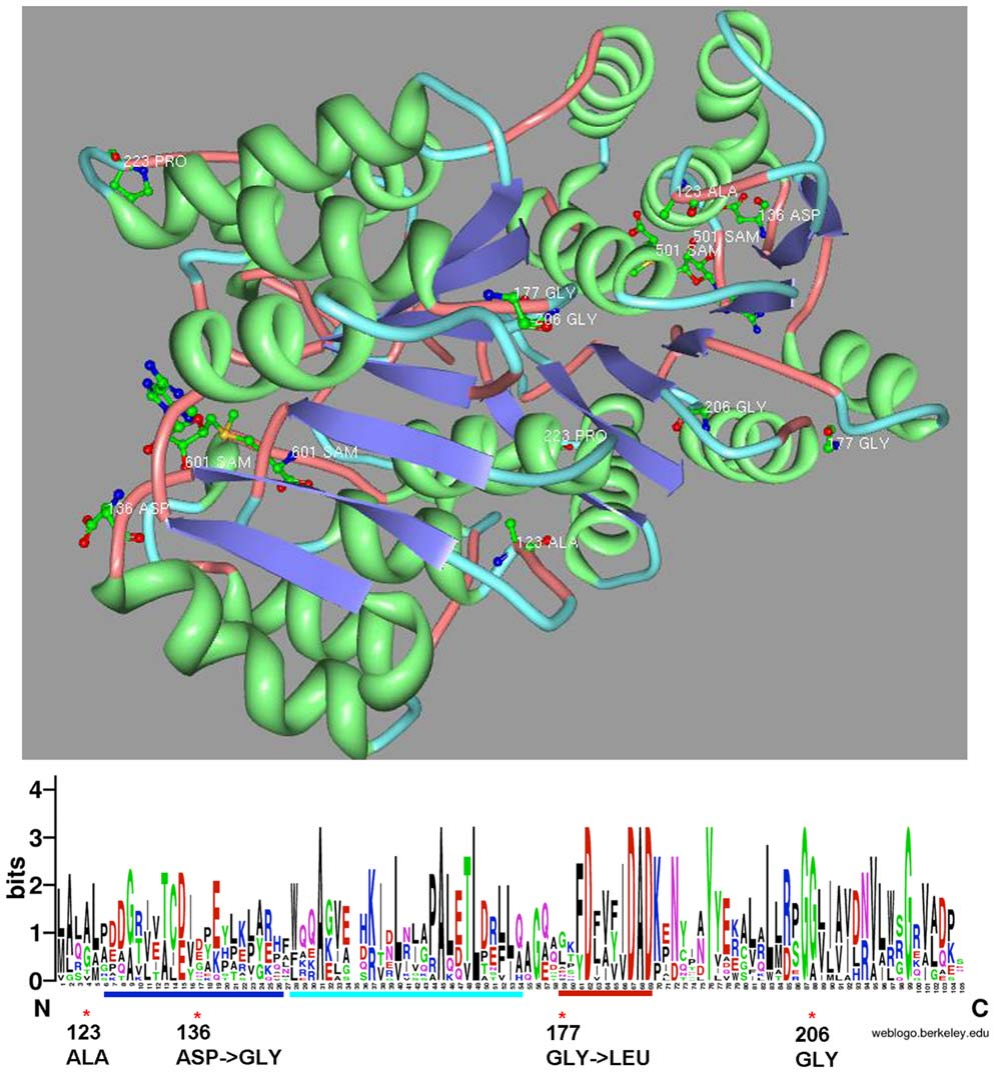
Motif conservation profiles and polymorphic sites map proximal to a 3-D region of the predicted o-methyltransferase active site. MEME motif regions are mapped against a Weblogo built with representative sequences (Figure 5). Non-synonymous polymorphisms in the o-methyltransferase protein identified in different subspecies of Francisella are indicated (*). The position relative to the human ortholog is given and cases where the amino acid is divergent between the Human and *F.t.* subspecies *tularensis* sequence is indicated below the motif. Key sites are spatially proximal to the o-methyltransferase active site (501 SAM) and in specific cases, mark the transition between a turn-coil region (123 ALA, 177 GLY). The labeled amino acid (223 PRO) is where the *F.t.* subspecies *holarctica* homolog truncates.

The effect of subspecies specific sequence differences on the predicted o-methyltransferase protein product was further analyzed using the JNet method to predict secondary structures based on the sequence profile of contiguous stretches of amino-acid sequence in the o-methyltransferase sequence alignments (Figure 3) [31]. A predicted structural profile of alternating α-helices and β-sheets characteristic of this family of conserved o-methyltransferases was identified in the predicted secondary structures of the Francisella Type A o-methyltransferase (EC 2.1.1.104) and highly conserved motif profiles were also identified (Figure 5). The solved structures for this family of Caffeoyl-CoA o-methyltransferases include two from plants, (1sui and 1sus), present in *Medicago sativa* (Alfalfa) and *Mesembryanthemum crystallium*. In both plant and human (PDB: 2AVD)(Figure 6), the protein consists of two distinct dimer subunits of approximately six β-sheets alternating with α-helices. Approximately three β-sheets denote a structural core region proximal to the active site of the protein.

Comparison of the plant and human structures to the predicted secondary structure of the Francisella o-methyltransferase variants shows that the *Francisella tularensis* subsp. *tularensis* subtype is structurally the most similar. Specifically, the core region of three β-sheets alternating with α-helices is conserved between the Human, *Francisella tularensis* subsp. *tularensis*, *Mycobacterium tuberculosis*, and *Coxiella burnetii* predicted o-methyltransferase protein structures and therefore, may functionally mimic the active site of the human host protein. This is supported by the identification of highly conserved sequence motif blocks (Figure 5) that are in the region of the predicted o-methyltransferase active site (Figure 6), and therefore, likely to be functionally relevant. In addition, protein structure mapping of distinct sequence changes identified as nonsynonymous variations in the Francisella o-methyltransferase orthologous family (Figure 2a) was done relative to the human ortholog (Figure 6) and occur in the three dimensional space surrounding the predicted active site, or in regions of a turn-coil transition (Figure 6).

Similar consensus sequence profiles in conserved regions of the o-methyltransferase proteins present in host genomes and the intracellular pathogens: *Coxiella burnetii*, *Francisella tularensis* and *Mycobacterium tuberculosis* is suggestive of a functional role for this gene family during infection. Coxiella is one of the closest bacterial relatives to the Francisella genus and overall, the *Coxiella burnetii* o-methyltransferase protein sequence is also phylogenetically closest in relation to the Human ortholog (Figure 1). *Coxiella burnetii* is the causative agent of Q fever, with broad and diverse affects on numerous mammals. In humans, the severity of disease varies and has the most profound impact on immunocompromised individuals and those working in agriculture since livestock, primarily cows and goats, harbor large *Coxiella burnetii* bacterial loads [32].

Additional analysis of the *F. tularensis* subsp. *tularensis* SchuS4 and *Mycobacterium tuberculosis* protein sequence alignments using Monte Carlo techniques (Figure S2) as well as a Neighbor Joining and Maximum Likelihood approach (data not shown) provide evidence of a closer relationship between the o-methyltransferase sequences identified in the most virulent strains of both *F. tularensis* and *M. tuberculosis*. These virulent, and in some cases multi-drug resistant, strains that exhibit the highest similarities include: *Mycobacterium tuberculosis* H37Rv, *Mycobacterium tuberculosis* F11 (ExPEC), *Mycobacterium avium* subsp. *paratuberculosis* K-10, and *Mycobacterium avium* 104. Outbreaks of Mycobacterium are typically caused by hypervirulent strains of *Mycobacterium tuberculosis*, the majority of which carry deletions in genes encoding products important for cell wall architecture or regulation, particularly in response to environmental cues. These pathogens also leverage protective niches within the host, such as granulomas, that facilitate pathogen evasion from host protective mechanisms and therefore, enable long-term and persistent infection [33]. *Mycobacterium tuberculosis* H37Rv is perhaps the most well known isolate of the Mycobacterium genus, and is the etiological cause of Tuberculosis. It is a highly pathogenic bacteria and widely distributed geographically. *Mycobacterium tuberculosis* F11 and *Mycobacterium tuberculosis* T46 are also highly virulent human pathogens. The *M. tuberculosis* F11 strain was isolated from patients in the Western Cape of South Africa during a TB epidemic and the *M. tuberculosis* T46 strain was isolated in 1996 from a patient in San Francisco [34]. *Mycobacterium avium* subsp. *paratuberculosis* K-10 (“Map”) is a gram-positive, slow-growing pathogenic bacteria that is the etiological cause of Johne's disease in cattle and there have been controversial implications that it may cause Crohn's disease in humans [34]. Similar to most Mycobacteria isolates, Map is difficult to treat and is not sensitive to drug regimens used to treat *M.tuberculosis*.

Although expression of the *Mycobacterium tuberculosis* H37Rv o-methyltransferase ortholog (Rv1220c) is not highly correlated with neighboring genes, it is proximal to a cluster of highly regulated genes encoding ABC transporters, a protein family known to play important roles in bacterial virulence [34]. Genes with the highest correlated expression with the *Mycobacterium tuberculosis* H37Rv (Rv1220c) gene, ranked by a Co-expression Coefficient (CexpC), encode predicted NADH dehydrogenase subunits N and L (CexpC = 0.55032 and 0.48066), penicillin-binding protein DacB1 (CexpC = 0.49191), membrane-associated phospholipase C (CexpC = 0.46973), UDP-N-acetylmuramate-L-alanine ligase (CexpC = 0.45189), and D-alpha-D-mannose-1-phosphate guanyltransferase (CexpC = 0.43689) [34]. Interestingly, there is a negative correlated expression of the Rv1220c gene with the DevR/DosR transcription factor (CexpC = −0.37535), which has been shown to mediate the genetic response of *Mycobacterium tuberculosis* to oxygen limitation and nitric oxide exposure important for the regulation of the latency stage of infection [34]. A negative correlation of expression with the DevR/DosR transcription factor suggests that the o-methyltransferase gene is likely important for regulating a stage of infection and virulence that is distinct from the latent phase of the pathogen's life cycle. Roles of methyltransferase proteins during infection of macrophages by *Mycobacterium tuberculosis* and *Mycobacterium avium* 104 have been described in other studies [35], [36]. In *Mycobacterium tuberculosis*, transient expression of two S-adenosylmethionine-dependent methyltransferases occurs during the initial phase of macrophage infection [35]. In *Mycobacterium avium* 104, methyltransferase D mediates the methylation of highly antigenic glycopeptidolipids (GPLs) found densely distributed on the cell surface [36].

Intracellular pathogens, such as *Mycobacterium*, *Coxiella*, *Legionella*, and *Francisella* have all adapted similar and complex mechanisms of evasion for survival in the host, including evasion of lysosome mediated degradation. Lysosome mediated degradation of invasive bacterial pathogens is a general mechanism of defense used by the host immune system. Prevention of fusion and degradation by the host lysosome is fundamental to mechanisms of evasion, and this allows for the bacteria to replicate within macrophage maturation-defective phagosomes. The molecular mechanisms mediating successful survival and replication of pathogens, as well as mechanisms of host protein inhibition or degradation, are largely unknown. Recent studies have provided significant insight into proteins that regulate the process of host-evasion. In *Legionella*, the Dot/Icm proteins of the Type IV secretion system regulate a mechanism of molecular mimicry that intercepts the host polyubiquination machinery, and ultimately inhibits phagosome-lysosome fusion [17]. Upon infection of host cells, many bacterial pathogens use Types III–VII secretion systems and translocation machinery to inject numerous effector proteins into host cells. One of the effector proteins introduced into the host cell in *Legionella* by the Dot/Icm Type IV secretion system, is an Ankyrin repeat protein (AnkB) that is required for docking polyubiquinated proteins to the *Legionella* -containing vacuole within the infected macrophage [17], [37]. Furthermore, Dot/Icm mediated recruitment of several proteins shown to be required for the function of AnkB are also necessary for intracellular proliferation of the pathogen. These proteins include farnesyltransferase, RCE-1 (Ras-converting enzyme-1), and iosprenyl cysteine carboxyl methyltransferase host farnesylation enzymes [17], [37]. It is evident that families of highly conserved methyltransferases play a role during infection of host macrophages by pathogenic bacteria; and in the case of *Legionella*, have a specific role in a molecular mechanism of mimicry that is necessary in order for the pathogen to evade host-defense responses.

That said, much remains to be understood regarding how shared mechanisms maybe regulated to reconcile subtle differences of pathogen potency, likely dependent on the host niche. For example, recent epidemiological studies have suggested that infections caused by isolates of a specific *F.t.* subspecies *tularensis* lineage, Type AI.b, result in significantly higher rates of mortality [38], [39]. These studies have reported that C57BL/6 mice challenged intradermally succumb to Type AI.b infection at significantly earlier times than Types AI.a or A.II infected mice, although a similarly low bacterial load in infected tissues post-mortum was reported for mice infected with either Types AI.a or AI.b strains [38]. As expected, bacterial loads measured in the lungs from mice infected with different Francisella subtypes differ dramatically depending on the route of infection (intradermally vs. aerosolization), and are likely indicative of different mechanisms of dissemination post-infection [40]. In addition, variation of clinical signs during the course of infection have been reported for different strains of mice (BALB/c vs. C57BL/6) [40]. Studies using different mouse strains and examining both modes of infection (intradermally vs. aerosolization), have reported that the greater virulence of the Type AI.b isolate (FSC033) in comparison to the Type AI.a isolate (SchuS4) is often subtle and for the most part, differences in virulence seem not to be biologically significant [41], [42]. Together, the findings detail a complex host-pathogen relationship that would require further studies of mechanisms of infection and dissemination, as well as immunological responses required to combat systemically-initiated versus inhalation-initiated tularemia. Although subtle differences, that in some cases are significant, may exist between distinct Type A lineages; there are likely mechanisms of infection that are highly conserved among the virulent Type A strains included in this comparison. Furthermore, the presence and conservation of orthologous gene families in otherwise very distant bacteria is indicative that shared mechanisms of molecular mimicry may be widely adopted by pathogenic bacteria exhibiting similar life-styles.

## Materials and Methods

### Identification of conserved o-methyltransferase orthologs

The pipeAlign analysis pipeline was used to search for orthologs of the *F. tularensis* subsp. *tularensis* o-methyltransferase protein [21]. Specifically, the protein sequence for the SchuS4 o-methyltransferase (YP_170657.1) was used to query a composite database of 13,062,032 sequences (4,215,264,833 total amino acids) using both a gapped and un-gapped blastp searches run with default filtering for comparison and validation purposes. The Ballast algorithm predicted 10 LMSs (Local Maximum Segments) covering 118 residues (54.1%) of the query sequence. The NorMD assessment value for the DbClustal alignment was 0.580 and after treatment of the DbClustal alignment by Rascal, the NorMD value was 0.584. The Leon algorithm was able to cluster the multiple alignment into distinct families (NorMD = 0.581), without removing any sequence and DPC distance matrix based clustering distributed the 200 sequences into 7 distinct groups. Duplicate protein sequences were manually removed from the output multiple sequence alignment. Francisella o-methyltransferase sequences with significant similarity are listed in Table 1.

**Table 1:** Francisella o-methyltransferase orthologs.

Locus Id	Species
FTMG_01264	*F. tularensis* subsp. *tularensis* MA00-2987
FTBG_01430	*F. tularensis* subsp. *tularensis* FSC033
FTT_1766	*F. tularensis* subsp. *tularensis* SchuS4
FTDG_01299	*F. tularensis* subsp. *novicida* GA99-3548
FTDG_01300	*F. tularensis* subsp. *novicida* GA99-3548
FTL_0091	*F. tularensis* subsp. *holarctica* LVS
FTHG_00122	*F. tularensis* subsp. *holarctica* 257
FTG_1138	*F. tularensis* subsp. *novicida* FTG

In addition, the *F. tularensis* subsp. *tularensis* SchuS4 o-methyltransferase protein sequence was used to query whole-genome bacterial sequence databases using blastp (BLOSUM62 matrix, default parameters with the exception of setting the expectation threshold to e<1e-11). Twelve predicted orthologs were identified belonging to different species of Mycobacterium, four representing paralogs present in the same strains. A total of 21 protein sequences (12 Mycobacterium and 9 Francisella) were used for clustalw and muscle analysis alignments at default settings. In the clustalw alignment shown (Figure S1), the minimum sequence length = 137 and maximum alignment length = 246, with an average length of 210 amino acids.

In order to assess if the alignments between *F. tularensis* subsp. *tularensis* SchuS4 and *Mycobacterium tuberculosis* o-methyltransferase sequences are significant, the alignments were evaluated using PAM scoring matrices (in Matlab). In addition, random permutations of the *F. tularensis* subsp. *tularensis* o-methyltransferase protein sequence were generated and global alignments of these random sequences with the Mycobacterium protein sequence was calculated using customized Matlab scripts, and supported a significant relationship. The statistical significance of the alignment scores to the random sequences was approximated using a type 1 extreme value distribution and a plot of the probability density function of the estimated distribution was generated in Matlab (Figure S2).

### Phylogenetic relationships of the o-methyltransferase protein families

Clustalw multiple protein sequence alignments of 212 o-methyltransferase orthologs were used to construct a phylogenetic tree using Maximum likelihood Estimation (PhyML, MEGA5) (Figure 1). The Maximum likelihood tree was constructed using the JTT matrix-based model with bootstrap values of 1000. The truncated Francisella tularensis subsp. novicida SPT_A7JP42_FRANO/1-83 sequence was removed since it was such an extreme outlier due to its limited homology to only the sequence region 5′ of the conserved Francisella MAQ consensus sequence. An approximate-maximum likelihood approach, FastTree, was also used to analyze the phylogenetic relationships specifically between the Francisella and Mycobacterium o-methyltransferases (Data not shown) [43]. Phylogenetic trees were inferred from alignments of the protein sequences using the BLOSUM45 amino acid distance matrix and the JTT(Jones-Taylor-Thornton) model of amino acid evolution. A single rate for each site (the CAT approximation) was used in order to account for varying rates of evolution across sites. The reliability of each split in the tree is estimated by computing local support values with the Shimodaira-Hasegawa test (similar to PhyML's SH-like local supports) [43]. There were 20/20 unique and 0/17 bad splits calculated.

### Analysis of predicted genome rearrangements, recombination, and selection

Whole genome alignments and dotplot comparisons are provided by the Broad Institute and the methodology for the Francisella comparative genome project is published [12]. Alignments of the o-methyltransferase coding sequences were analyzed for recombination using the PHI test in SplitsTree v4 [28]. The algorithm identifies cases where a single sequence exhibits similarity to multiple, other sequences at different points along its length as evidence of phylogenetic ambiguity within the alignment. The PHI test was run using a window size of 100 with k as 45. A multiple sequence alignment of the Francisella o-methyltransferase homologous sequences (representative of each subspecies variant) was done at the amino acid level using MUSCLE [30], [44] with default parameters. The resulting protein alignments were converted into codon-based nucleotide alignment using PAL2NAL [30], [45]. The codon-based alignment was analyzed on a per-codon basis using several NSsites models available using codeml/PAML for positions under positive selection [30]. Selection analysis was also done using several algorithms that are part of the HyPhy package [46].

### O-methyltransferase secondary structure predictions

JNET secondary structure prediction was done using the Jpred 3 secondary structure prediction server at default settings. A clustalw multiple sequence re-alignment was performed for building a Hidden Markov Model (HMMer 2.3.2) profile of 218 amino acid sequence (Ave accuracy = ∼80%).

### Conserved motif identification and site mapping to the human o-methyltransferase protein structure

Orthologous o-methyltransferase protein sequences representative of the Francisella, Coxiella, Mycobacterium, Human, Rat and Mouse subtypes were aligned using MUSCLE [44] and visualized with WebLogo [47]. A sub-alignment of approximately 70 amino acids with gaps removed was also analyzed using MEME with small-sample correction (SSC) [48]. Sites identified as nonsynonymous variations were mapped to the WebLogo alignment and the human protein structure using PDB ProteinWorkshop 3.9.

## Supporting Information

Figure S1 **Multiple alignment of significantly similar Francisella and Mycobacterium o-methyltransferase protein sequences.** Blastp search identified Mycobacterium o-methyltransferase sequences exhibiting a significant overall similarity to Francisella. Several conserved sequence blocks are identifiable throughout the alignment and were included in building the conserved motif profiles (Figure 5).
(TIF)

Figure S2 **Relative Evolutionary Distance Between Francisella and Mycobacterium.** Significance of sequence alignments between Francisella and Mycobacterium o-methyltransferase proteins was done using Monte Carlo techniques. Specifically, *Francisella tularensis* subsp. *tularensis* SchuS4 random sequence permutations were aligned to A) *Mycobacterium tuberculosis* H37Rv (p distribution = 0 and score = 86) and B) *Mycobacterium tuberculosis* CDC1551 (p distribution = 0 and score = 84.34). A statistical significance of the alignment scores to the random sequences was approximated using a type 1 extreme value distribution and a plot of the probability density function of the estimated distribution is shown to be more significant in A) than B).
(TIF)
