# Supplementary figures and images for: Host-Pathogen O-Methyltransferase Similarity and Its Specific Presence in Highly Virulent Strains of Francisella tularensis Suggests Molecular Mimicry
Source: PLoS One. 2011 May 26;6(5):e20295. doi: 10.1371/journal.pone.0020295 (PMC3102702; doi:10.1371/journal.pone.0020295)

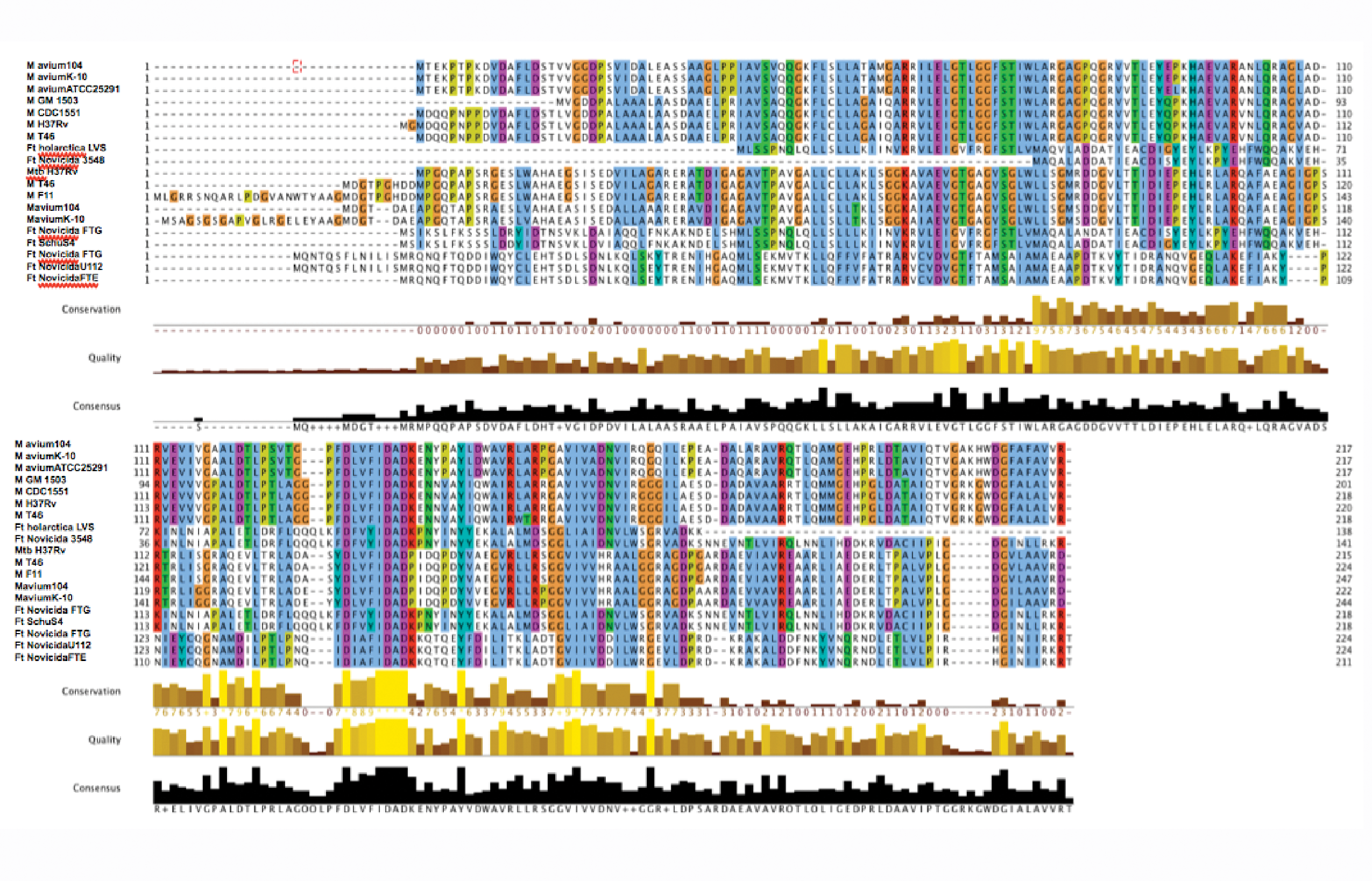

Supplement: Figure S1 — Multiple alignment of significantly similar Francisella and Mycobacterium o-methyltransferase protein sequences. Blastp search identified Mycobacterium o-methyltransferase sequences exhibiting a significant overall similarity to Francisella. Several conserved sequence blocks are identifiable throughout the alignment and were included in building the conserved motif profiles (Figure 5). (TIF) [file pone.0020295.s001.tif]

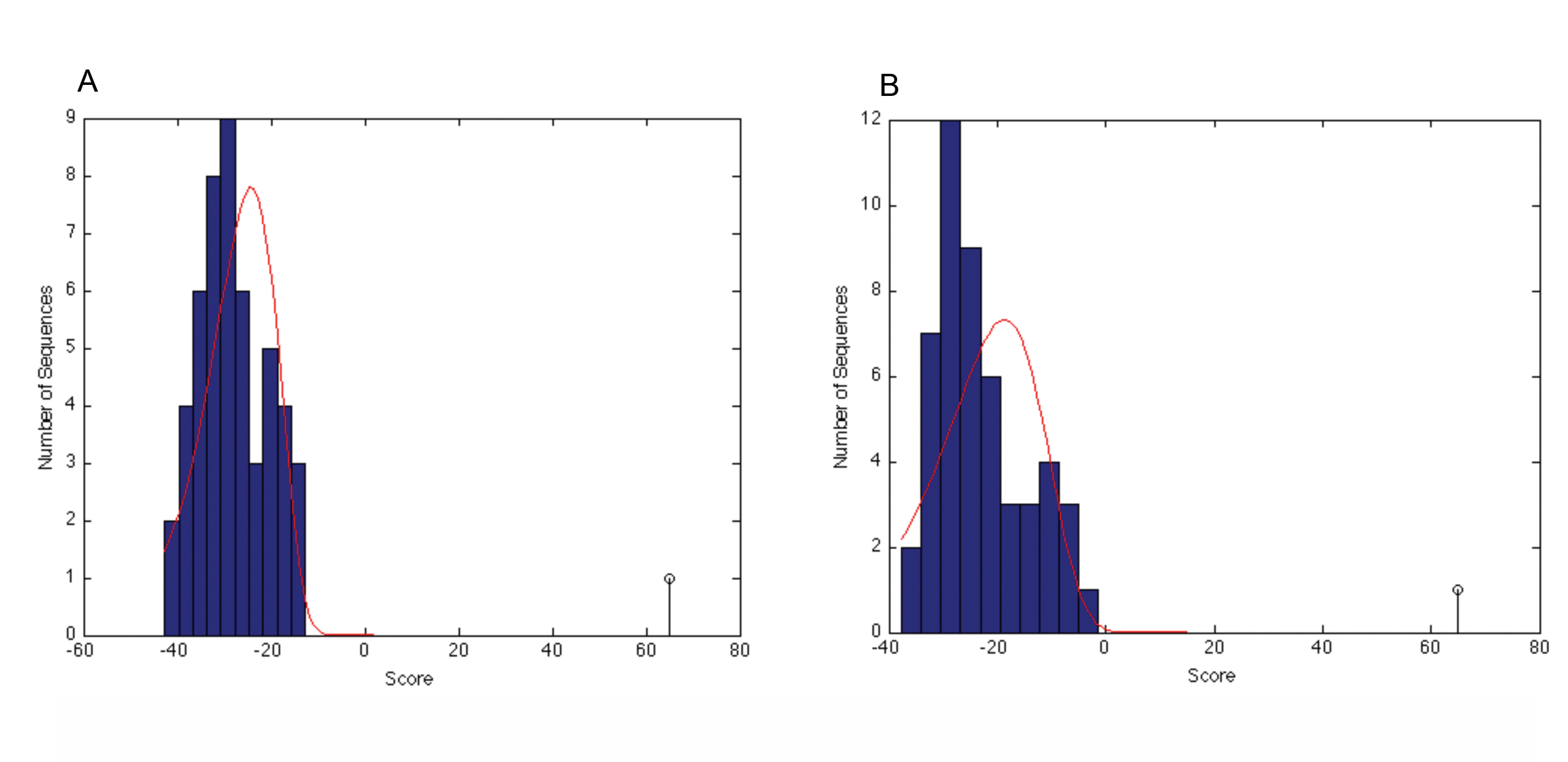

Supplement: Figure S2 — Relative Evolutionary Distance Between Francisella and Mycobacterium. Significance of sequence alignments between Francisella and Mycobacterium o-methyltransferase proteins was done using Monte Carlo techniques. Specifically, Francisella tularensis subsp. tularensis SchuS4 random sequence permutations were aligned to A) Mycobacterium tuberculosis H37Rv (p distribution = 0 and score = 86) and B) Mycobacterium tuberculosis CDC1551 (p distribution = 0 and score = 84.34). A statistical significance of the alignment scores to the random sequences was approximated using a type 1 extreme value distribution and a plot of the probability density function of the estimated distribution is shown to be more significant in A) than B). (TIF) [file pone.0020295.s002.tif]
